# Reports of Cases Occurring in the Medical Ward of the Buffalo Hospital

**Published:** 1857-12

**Authors:** A. W. Nichols

**Affiliations:** Assistant to Chair of Clinical Medicine, University of Buffalo


ART. II.—*Abstract of the Clinical Lectures delivered at the Buffalo Hospital of the Sisters of Charity, upon the Diseases treated in the Male Medical Ward, by* AUSTIN FLINT, M. D., *Attending Physician, during the present College Session, viz., from October* 14*th,* 1857, *to February* 27, 1858. Reported by A. W. NICHOLS, M. D., Assistant to Chair of Clinical Medicine, University of Buffalo.

No. I. *For the Month of October.*

*Typhoid Fever; Treatment; Spasmodic Respiration and Apoplectic Coma; Delirium during course of.*

Two cases of typhoid fever, in the ward, illustrated the phenomena of this disease in its earlier and later stages. In one patient, delirium has just manifested itself. In the other, the delirium is observed only occasionally, and at night. In the former case, the delirium is active, consisting in incoherent talking and repeated attempts to get out of bed. Besides this symptom, which is now observed only at night, the mental faculties appear to be blunted, the countenance is inexpressive; aspect dull; there is slight cough and expectoration, with sibilant and sonorous râles on auscultation, and a moderate degree of febrile movement. In the latter case, which is now approaching the period of convalescence, the delirium, which a few days since was active and noticed during the greater part of the time, is now observed only occasionally at night, and consists of incoherency in talking; the intellect is brighter; the countenance more expressive; the pulse diminished in frequency and increased in volume.

Delirium is a very common and a very prominent symptom in this form of fever. It naturally excites alarm with the patient's friends; and they not only grow solicitous about his immediate condition, but frequently entertain apprehensions in regard to the ultimate recovery of his intellect. But, in the great majority of instances, after the fever has pursued its allotted course, delirium disappears, and the patient regains full possession of his intellectual faculties. We can, therefore, ensure the friends of the patient, of the transitory duration of this symptom.

In this connection, the case of the person who is now convalescing, and who, as above mentioned, was actively delirious during the career of the fever, is worthy of notice. He has been free from any aberration of mind, during the past two clinical days. But, the other night, incoherent talking on various subjects, was observed by the nurse. Since then, no farther manifestation of delirium has been observed; and convalescence is progressing rapidly.

These occasional attacks of delirium occurring during convalescence, should not be regarded as relapses nor even as showing a tendency to relapse. True relapses in this variety or form of continued fever, are exceedingly rare. Prof. F. has met with but a single instance of the kind, in all the time the disease has been under his observation. In this case, after the fever had pursued its course and exhibited many of its ordinary and characteristic phenomena, and convalescence had been established, the patient was again subjected to a second attack of the fever, passing through its usual stages as though he had not had the disease previously

*The treatment* of typhoid fever as pursued in the hospital, consists in sustaining measures, and the alleviation of any unpleasant symptoms which may arise during the course of this disease. The sustaining measures are diffusible stimulants, as brandy or whisky, given in considerable doses and quite undiluted, alternating every two hours with milk porridge and the concentrated juice or essence of fresh meats, such as beef. These measures constitute the *sustaining diet* of the hospital. Hitherto, brandy has been employed generally, but the difficulty in obtaining a pure article, at a reasonable price, has led Prof. F. to the use of whisky, and this is found to answer as well the purposes for which brandy was formerly prescribed.

In the patient who is now convalescing from typhoid fever, a somewhat unusual, and at first view, strange measure was put in practice, several days since, during the height of the fever. The patient had presented for some time previous to this, distension of the abdomen, with tympanites, accompanied with frequent liquid evacuations passed in bed. The patient was actively delirious. His pulse was not small, nor was it much accelerated. Prof. F. regarding the diarrhœa as an effort of the system to relieve the intestines of their contents, directed a *saline cathartic* to be given: magnesiæ sulph., ʒj, every two hours until the evening, this being about noon. The following morning, the nurse stated that two doses of the above medicine had been taken, and a copicus dejection occurred during the night. Since then, and for three days thereafter, no dejection was observed. There was less distension of the abdomen, and also less tympanites; and an improvement in the condition of the patient generally.

The practice of giving saline cathartics in typhoid fever, originated with the French, who place much reliance in them in cases similar to the foregoing.

The patient who was noticed on the first clinical day as being in the earlier stage of typhoid fever, presents, this morning, a symptom indicative of great danger, namely, what is known as *spasmodic respiration;* the inspiration being shortened and hurried. This was observed last evening for the first, and is not quite as marked this morning; but the patient is now becoming comatose and cannot be roused. He lies with his eyes closed, uttering no noise, and making no movement. This condition has been termed *apoplectic coma.* Spasmodic respiration precedes for a greater or less length of time, this comatose state; and when observed, we should make use of every means in our power to avert the impending danger. Prof. F. had pointed out some years since the connection between these two conditions, and the result of his observations will be found in his Report on Continued Fever, page 197, et. al.

### *Intermittent Fever; Treatment.*

There were several cases of this form of fever in the hospital, and it would be noticed that they were treated very much alike.

In the treatment of intermittent fever, there were three indications usually: 1st. To arrest the paroxysms as speedily as possible; 2d. To continue the remedy so as to prevent their return; and 3d. To relieve the coëxisting anæmia. Quinia was the medicine used in the hospital. Large doses of the sulphate of quinia, say five grs. three times daily, were given immediately, without reference to any coëxisting complaint. In a day or two, usually, the paroxysms were arrested; and then, it was found the dose of the remedy could be diminished rapidly, so that but a grain or two, twice daily, would serve to prevent a return of the paroxysm. Whenever anæmia existed, a ferruginous tonic was added; the sulphate of iron, or citrate of iron and quinia, being commonly employed.

A case of what is known as *latent intermittent*, was presented to the notice of the class. Here, some of the stages of the paroxysms are deficient; or it recurs at irregular intervals. A similar plan of treatment is adopted in such cases.

### *Prurigo.*

A case now in the ward, presents the characteristic appearances of this affection, well-marked. Two, and sometimes three, species of cutaneous diseases, are mentioned by writers as belonging to the order of papulæ or pimples, namely, lichen, strophulus, and prurigo. Lichen and strophulus may be considered as varieties of the same species. Prurigo differs from lichen in the following particulars: the pimples are larger, pyrammidal, and flattened, instead of acuminated, and not reddened. As a general rule, the eruption is seated on the anterior part of the extremities. The pimples are colorless, and, therefore, not easily seen; they are felt better, by pressing the hand over the surface. The pruritus is severe, greater at night, and prevents sleep. Scratching does not relieve the pruritus, but excites it. The scratching occasioned by the pruritus, give rise to the presence of minute black scabs; this appearance is highly characteristic of prurigo.

### *Intercostal Neuralgia.*

In a patient who had just been admitted, there was presented a very good example of intercostal neuralgia. This man complained of pain, quite sharp, in the lower and lateral portion of the left side of the chest, which was increased by forced breathing and by the act of coughing. On inspection of the chest, the expansive movement was instinctively restrained, the patient being unaware of any difference of expansion on the two sides of the chest. Loss or want of mobility on one side of the chest was found to exist in the course of various intra-thoracic diseases, as well as in affections of the parietes of the thorax. When this physical sign is present in intercostal neuralgia, we must be careful not to attribute it to intra-thoracic disease, such as pneumonitis or pericarditis; nor to other diseases of the walls of the thorax, as pleuritis; nor to paralysis of the respiratory muscles of the chest. These latter diseases will easily be recognized usually by recourse to a physical examination of the chest; and no difficulty would attend the diagnosis of paralysis of the muscles. Cough and expectoration were noticed in this case; but the diagnosis was rendered plain by pressing over the spine close to the spinous processes, laterally to the outside of the nipple, and anteriorly, close to the edge of the sternnm. On the right side, over these portions of the thorax, no tenderness was elicited; on the left side, tenderness existed quite marked, in two or three of the intercostal spaces to the left of nipple and below it, and close to the edge of the sternum, and over the middle dorsal vertebræ. This disease is frequently mistaken for pleurodynia, but the above sign, namely, tenderness limited to three points on one side of the thorax, is characteristic of the former affection.

The *treatment* of intercostal neuralgia consists essentially in the employment of tonics and anodynes, of which quinia and morphia seem to be the most appropriate. For further information on this disease, vide Buff. Med. Journ., for July, 1857.

*Pulmo-Tuberculosis.*

The *pleuritic pains* attending the course of phthisis, are to be distinguished from rheumatic or neuralgic pains. The former are stitch-like and lancinating, and increased more by forced respiration than by movements of the body or upper extremities; and, besides, they are not confined to particular points on the thoracic parieties, nor diffused over any considerable portion of the chest; but, usually, are present at the summit of the lung. These pleuritic pains indicate the existence of a certain amount of inflammation of the pleura. This variety of pleuritis accompanying the course of pulmo-tuberculosis, is called *dry* pleuritis, on account of the absence of liquid effusion. The pleuritis also is circumscribed, and is due to the presence of tubercles disseminated through the pulmonary tissue. The pleural surfaces become united, and thus the pleuritis proves conservative. For, as the tuberculous matter passes through its successive stages and is expelled, the union of the surfaces of the pleural membrane, prevents the evacuation of the tuberculous matter into the cavity of the pleura. That this accident does in reality occur, is well known. When tuberculosis, departing from the law of location, attacks the lower lobe of the lung, instead of the summit, the whole lobe being in a short period rendered almost useless, ulceration and perforation of the visceral layer of the pleura occur in some instances; and the disease called pneumo-hydro-thorax is produced, which very frequently proves fatal.

A patient in the ward, affected with pulmo-tuberculosis, was observed to speak with difficulty; his voice was husky; he could scarcely articulate in fact. He presented an affection of the larynx not infrequently met with in the course of pulmo-tuberculosis. This was laryngitis, or laryngeal phthisis as it was formerly called. Up to a few years ago, it was thought that tuberculosis first attacked the larynx, and then proceeded downward to the pulmonary tissue; and hence the name, laryngeal phthisis. Subsequent researches have disproved this opinion, and established, beyond a doubt, that tuberculosis first attacks the lungs, and after a greater or less period of time other organs, as the larynx, may become affected. If the former opinion had been correct, it would have been of very great importance to have arrested the disease, if practicable, whilst the larynx alone was affected. But, at the present time, knowing that tuberculosis has already invaded the lungs, this laryngeal complication becomes of far less importance. Not infrequently there exists complete loss of voice or aphonia, and ulceration of the structure of the larynx.

